# Magnetization switching by asymmetric topological surfaces

**DOI:** 10.1093/nsr/nwaf178

**Published:** 2025-05-09

**Authors:** Zihan Li, Sheng Pan, Shanshan Liu, Yuda Zhang, Ang Li, Linfeng Ai, Xiaoyi Xie, Xiangyu Cao, Zehao Jia, Xiaodong Han, Jiexiang Yu, Faxian Xiu

**Affiliations:** State Key Laboratory of Surface Physics and Department of Physics, Fudan University, Shanghai 200433, China; School of Physical Science and Technology, Soochow University, Suzhou 215006, China; State Key Laboratory of Surface Physics and Department of Physics, Fudan University, Shanghai 200433, China; State Key Laboratory of Surface Physics and Department of Physics, Fudan University, Shanghai 200433, China; Beijing Key Laboratory of Microstructure and Properties of Advanced Materials, Institute of Microstructure and Properties of Advanced Materials, Beijing University of Technology, Beijing 100124, China; State Key Laboratory of Surface Physics and Department of Physics, Fudan University, Shanghai 200433, China; State Key Laboratory of Surface Physics and Department of Physics, Fudan University, Shanghai 200433, China; State Key Laboratory of Surface Physics and Department of Physics, Fudan University, Shanghai 200433, China; State Key Laboratory of Surface Physics and Department of Physics, Fudan University, Shanghai 200433, China; Beijing Key Laboratory of Microstructure and Properties of Advanced Materials, Institute of Microstructure and Properties of Advanced Materials, Beijing University of Technology, Beijing 100124, China; School of Physical Science and Technology, Soochow University, Suzhou 215006, China; State Key Laboratory of Surface Physics and Department of Physics, Fudan University, Shanghai 200433, China; Institute for Nanoelectronic Devices and Quantum Computing, Fudan University, Shanghai 200433, China; Shanghai Research Center for Quantum Sciences, Shanghai 201315, China; Zhangjiang Fudan International Innovation Center, Fudan University, Shanghai 201210, China

**Keywords:** spin-orbit torque, intrinsic magnetic topological material, asymmetric topological surfaces, field-free switching

## Abstract

The development of spin-orbit-torque (SOT) devices has sparked considerable research interest, particularly in the quest for novel materials that exhibit high spin-to-charge conversion efficiencies for effective magnetic switching. However, optimizing structure and improving efficiency necessitate theoretical insights and material innovations. In this study, we employ first-principles calculations to investigate the persistent spin current in magnetic topological material MnSb_2_Te_4_. The weakly asymmetric topological surface states ensure that the spin currents in this system do not cancel out while facilitating high spin-to-charge conversion efficiency through spin-moment locking, thus unprecedentedly enabling SOT switching within a single layer. In experiments, we demonstrate a low critical current density of 7.3 × 10^5^ A/cm^2^ for switching in epitaxial MnSb_2_Te_4_ thin films, alongside a substantial SOT efficiency of ∼41 at 6 K, consistent with micromagnetic simulations. Additionally, the development of *in-situ* epitaxial heterostructures of MnSb_2_Te_4_/FeTe_0.9_ allows for the extraction of an exchange-bias-induced effective field, thereby enabling field-free SOT switching within these heterostructures.

## Teaser

We demonstrate a current-induced magnetization switching originated from weakly asymmetric topological surface states, without the need for injected spin current, in epitaxial MnSb2Te4 thin films. Additionally, we observe field-free switching in MnSb2Te4/FeTe0.9 heterostructures.

With the continuous increase in demand for storage capacity and speed, manipulating the magnetization of spintronic devices by electric currents instead of external magnetic fields opens a wide spectrum of opportunities to reduce dimensions and energy consumption [1–3]. Spin-orbit torques (SOTs), as an effective electric switching approach, have great potential for breakthroughs both in spintronic theory and in device designs. Because of the nature of the spin-orbit coupling mechanism, the generation of spin currents usually requires specific materials and structures, such as a heavy metal/ferromagnet (HM/FM) system [4,5] or a specifically designed interface with broken inversion symmetry [6–8]. It seems that high spin-to-charge conversion efficiency and simplified structures with good stability and repeatability cannot be satisfied simultaneously. With enormous efforts devoted to theoretical study and materials research, high-efficiency SOT in topological insulators (TIs)/FM, antiferromagnetic systems, and field-free SOT can be respectively achieved [9–11]. CuMnAs is a remarkable antiferromagnet with a symmetry-breaking magnetic structure and a unique energy band [11,12]. Although the total spin accumulation in CuMnAs is zero due to the inversion symmetry breaking, the SOTs on two sublattices with opposite moments promote each other instead of canceling out. The electric switching of Néel vector can be realized without an auxiliary functional layer.

Inspired by the antiferromagnet CuMnAs, a magnetic system with novel band structures can serve as a simplified device that integrates spin generation and magnetic switching. Topological insulators, with their spin-moment locking [13–20], have the potential to combine high spin-to-charge conversion efficiency and exotic magnetic properties. Considering the magnetic structure, intrinsic magnetic TIs can be a platform for the exploration of new SOT systems [21–24]. Here, we investigate the SOT-driven magnetization switching in ferromagnetic MnSb_2_Te_4_ (MST) with corresponding theoretical analysis. MST has a layer structure similar to the antiferromagnetic TI MnBi_2_Te_4_ (MBT), which has been reported to have a stable crystal structure and layer-dependent topological bands [22,23]. Each layer of MST is a septuple layer (SL) composed of Te-Sb-Te-Mn-Te-Sb-Te in a triangular lattice [25–29]. Owing to the smaller difference in ionic size and electronegativity between Mn and Sb compared to the difference between Mn and Bi in MBT, MST materials tend to have a higher number of Mn-Sb antisite defects [28]. Therefore, there can be ferromagnetism and topological non-trivial bands in the MST system [26,30].

In traditional magnetic materials without neighboring layers, the generation of SOT is hindered by low spin-to-charge conversion efficiency and the same polarization direction. MST emerges as a compelling candidate for spintronic devices, as it exhibits both topological surface states and a site-mixing magnetic structure, suggesting the potential for significant spin currents. However, critical questions remain regarding whether these spin currents are sufficiently robust to generate SOT, and what mechanisms govern the interactions between spin currents and magnetic moments in this unique single magnetic topological insulator. Moreover, MST serves as an exceptional platform for exploring the relationship between spin configurations and asymmetric topological properties, a connection that has yet to be fully investigated within the context of SOT research.

## SPIN CURRENTS IN MnSb_2_Te_4_

**Figure 1.**
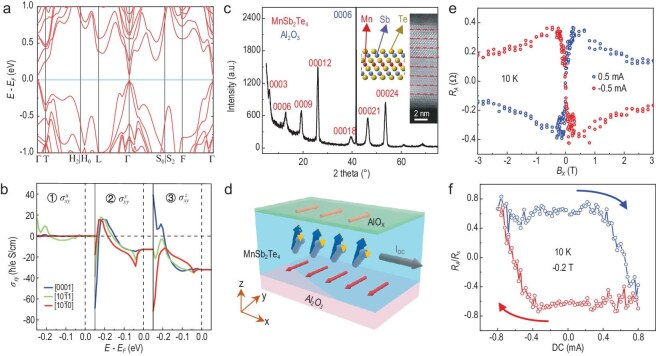
Spin-orbit-torque-induced magnetization switching in MnSb_2_Te_4_. (a) Electronic band structure of rhombohedral FM MnSb_2_Te_4_ with SOC. The magnetization is along the out-of-plane $+ z$ direction. The inequivalent time-reversal-invariant *k*-points are ${\mathrm{\Gamma }}( {0,0,0} )$, $T( {\pi ,\pi ,\pi } )$, $L( {\pi ,0,0} )$ and $F( {\pi ,0,\pi } )$. The valence band maximum is set to 0 eV. (b) SHC $\sigma _{xy}^{( {x,y,z} )}$ of bulk FM MST for different spin configurations. 1–3: Spin polarization along $x,{\mathrm{\,\,}}y,{\mathrm{\,\,}}z$ directions, respectively. Different lines indicate the Mn atomic spin configurations along the $[ {0001} ]$, $[ {10\bar 11} ]$ and $[ {10\bar 10} ]$ directions, respectively. The vertical dotted line represents the valence band maximum. (c) The XRD spectrum and cross-section TEM of the MnSb_2_Te_4_ film. The septuple atomic layers are marked with dashed lines. The thickness of the film is ∼10 nm. (d) Schematic representation of SOT-induced switching in MST. (e) Field-driven magnetization switching of the Hall bar device with ±0.5 mA dc biases at 10 K. (f) Current-induced switching under −0.2 T at 10 K. (e) and (f) display different magnetization states induced by dc currents, confirming the spin-torque mechanism instead of the Joule effect.

First, we perform theoretical calculations to investigate the overall spin currents derived from the band structure. Utilizing density functional theory (DFT), we computed the band structure of rhombohedral bulk ferromagnetic MST, incorporating SOC. As illustrated in Fig. 1a, a direct gap of ∼0.06 eV emerges at the ${\mathrm{\Gamma }}$ point. The spin Hall conductivity (SHC), denoted as $\sigma _{\alpha \beta }^\gamma $, is derived as a function of the energy using the Kubo formulas [31]:


(1) \begin{eqnarray*}
\sigma _{\alpha \beta }^\gamma = - \frac{{{e^2}}}{\hbar }\frac{1}{{V{N_{\boldsymbol k}}}}\mathop \sum \limits_n \mathop \sum \limits_k {f_{n{\boldsymbol k}}}{\mathrm{\Omega }}_{n,\alpha \beta }^\gamma \left( {\boldsymbol k} \right),
\end{eqnarray*}

where


(2) \begin{eqnarray*}
&&\Omega _{n,\alpha \beta }^{\textit{spin}\,\,\gamma }\left({\boldsymbol k} \right)\\
&& = - {\hbar ^2}\mathop \sum \limits_{m \ne n} \frac{{2Im\left|\big{\langle} {{\psi _{n{\boldsymbol k}}}{\mathrm{|}}\hat j_\alpha ^\gamma {\mathrm{|}}{\psi _{m{\boldsymbol k}}}\big{\rangle}\big{\langle}{\psi _{m{\boldsymbol k}}}{\mathrm{|}}{{\hat v}_\alpha }{\mathrm{|}}{\psi _{n{\boldsymbol k}}}} \big{\rangle}\right|}}{{{{\left( {{\varepsilon _{n{\boldsymbol k}}} - {\varepsilon _{n{\boldsymbol k}}}} \right)}^2} - {{\left( {\hbar \omega + i\eta } \right)}^2}}}.\\
\end{eqnarray*}

In these equations, ${f_{n{\boldsymbol k}}}$ represents the Fermi-Dirac distribution function, *n* is the band index, ${\varepsilon _{n{\boldsymbol k}}}$ denotes the energy eigenvalues, *V* is the primitive cell volume and ${N_{\boldsymbol k}}$ is the number of ${\boldsymbol k}$ points in sampling the Brillouin zone (BZ). The spin current operator $\hat j_\alpha ^\gamma {\mathrm{\,\,is\,\,\textit{expressed}\,\,as}}$  $\frac{1}{2}\{ {{{\hat s}_\gamma },{{\hat v}_\alpha }} \}$, where the spin operator ${\hat s_\gamma } = $  $\frac{\hbar }{2}{\hat \sigma _\gamma }$ and ${\hat v_\alpha }$ is the velocity operator. The SHC $\sigma _{\alpha \beta }^\gamma $ signifies that the spin current along the $\alpha $ direction is induced by the electric field along the $\beta $ direction, where the spin current is polarized along the $\gamma $ direction.

We calculated the SHC for three distinct spin configurations: magnetization along the +*z* direction($[ {0001} ]$), at a $45^\circ $ angle to the +*z* direction($[ {10\bar 11} ]$), and along the x direction($[ {10\bar 10} ]$). As shown in Fig. 1b, the SHC is constantly non-zero within the gap for $\sigma _{xy}^z$ and $\sigma _{xy}^y$. Notably, the magnitude of the SHC is invariant across different spin configurations. In contrast, the anomalous Hall conductivity (AHC) in Fig. S1, derived from Berry curvature, is zero for all three configurations within the gap. This behavior arises because the contributions of the valence band maximum and the conduction band minimum near the Fermi level primarily come from the non-magnetic Te($5p$) and Sb($5p$) orbitals, respectively.

The spin current is characterized as a magnetization-independent constant, which facilitates the generation of a persistent SOT. This finding is corroborated by experimental results. Utilizing molecular beam epitaxy (MBE), we successfully synthesized high-quality MST thin films on two-inch sapphire substrates. Figure 1c displays the X-ray diffraction (XRD) spectrum, revealing diffraction peaks corresponding to the (0001) orientation of the as-grown MST films. The lattice constant *c* is determined to be 41.09(3) Å, consistent with the bulk values. High-resolution transmission electron microscope (HRTEM) measurements were conducted to characterize the crystal structure, as illustrated in the inset of Fig. 1c. The SLs are marked with the corresponding atomic structures on the left. Based on the HRTEM analysis, the film thickness is estimated to be ∼10 nm, comprising 8 SLs. Magnetization measurements of the ferromagnetic MST films were performed using a superconducting quantum interference device (SQUID) system, as displayed in Fig. S3c. Both Zero-field-cooled (ZFC) and field-cooled (FC) curves show the paramagnetic-ferromagnetic transition below 25 K, allowing us to estimate the Curie temperature to be around 22 K.

To investigate the SOTs in MST films, micrometer-size Hall bar devices were fabricated, as shown in Fig. S3d. The channel width is 10 μm. In ferromagnetic materials, Hall resistances can be expressed as ${R_{xy}} = {R_{NH}} + {R_A}$, where *R_NH_* represents the normal Hall resistance and *R_A_* stands for the anomalous Hall resistance. By applying currents along the *x-*axis and magnetic fields in the *x*-*z* plane, the anomalous Hall effect (AHE) provides insights into the magnetization states and magnetic anisotropy. Angle-dependent AHE measurements in Fig. S3e demonstrate an out-of-plane easy axis in MST.

## SOT SWITCHING OF MnSb_2_Te_4_ WITHOUT EXTERNAL SPIN GENERATORS

By applying direct currents, we demonstrate that SOTs can drive the magnetization switching without the necessity for a multilayer structure, as shown schematically in Fig. 1d. To investigate the efficacy of SOTs in switching the ferromagnetic MST under in-plane fields, we employed various scanning modes at 10 K. As shown in Fig. 1e, we applied ±0.5 mA direct current (dc) biases during the field-dependent measurements. With a 0.5 mA dc bias, the AHE resistance *R_A_* transitions from negative to positive as the applied in-plane field changes from −3 T to 3 T, exhibiting the same polarity as the AHE without a dc bias. Conversely, with a −0.5 mA dc bias, the *R_A_* displays the opposite magnetization behavior as the field varies. In Fig. 1f, we swept the dc current from −0.8 mA to 0.8 mA, and then from −0.8 mA to +0.8 mA, with the in-plane field fixed at −0.2 T. We use the *R_A_/R_s_* to show the switching behavior, where the *R_s_* is the saturated AHE resistances. The normalized *R_A_* shows a clockwise loop with two distinct states that remain well-preserved at zero current. The critical switching current can be achieved at 0.73 mA, corresponding to a current density of 7.3 × 10^5^ A/cm^2^. The switching power consumption is 1.69 mW when the critical current is 0.73 mA, which is lower than that of some traditional devices. The critical current in MST devices is not only significantly smaller than the ∼10^7^ A/cm² in traditional HM/FM systems [32,33], but also lower than the ∼10^6^ A/cm² in other topological systems [34,35]. Moreover, the switching power is ∼1.5 W in a Hall-bar device based FePt/MgO system [32], much higher than the 1.69 mW in MST. Despite different working temperatures, MST shows great device performance among massive SOT systems. These observations indicate that in-plane fields and dc currents can independently modulate the different stable magnetization states in MST, which aligns with the SOT scenario based on damping-like torque [13,18].

**Figure 2.**
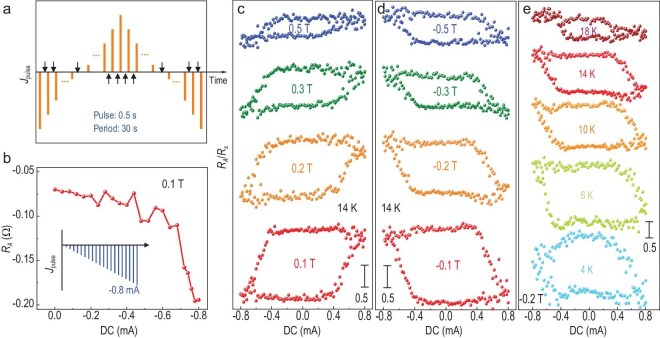
Current-driven switching behaviors in MnSb_2_Te_4_ at different temperatures and fields. (a) A schematic of the measurement procedure. After the large dc pulse current injection, indicated by the bar, the Hall resistance is measured under a low ac current of 10 μA. The magnitude of the pulse changes gradually from −0.8 mA to 0.8 mA, and returns to −0.8 mA. The width and period of the pulse are 0.5 s and 30 s, respectively. (b) The magnetization process induced by dc pulses after the application of an in-plane field of 0.1 T. The anomalous Hall resistance is saturated at −0.78 mA. (c, d) Current-induced switching at 14 K under in-plane fields varying from ±0.1 T to ±0.5 T. The switching polarity is anticlockwise and clockwise for positive fields and negative fields, respectively. (e) Current-driven switching under −0.2 T at 4 K to 18 K. The data at 10 K is the same as that in Fig. 1f. The saturated anomalous Hall resistances decrease with the elevated temperature.

Notably, the coercive field *H*_c_ in Fig. 1e is significantly reduced compared to measurements without dc currents, which can be attributed to the Joule heating effect [13,36,37]. To mitigate this effect, we employed a direct approach using current pulses while measuring *R_A_* with ac current in the dc-scanning mode. The pulse currents were applied with gradually changing magnitudes, as shown in Fig. 2a. The period and width of the pulse are 30 s and 0.5 s respectively, and the *R_A_* signals are collected at ∼25 s during each period. Although the pulse method helps reduce the Joule heating effect, it is important to exercise caution as currents exceeding 1 mA can cause irreversible damage to our devices. Prior to each measurement loop, the magnetizations are polarized by the in-plane field and current pulse. In Fig. 2b, after applying an in-plane field of 0.1 T, *R_A_* exhibits a negative value as the current changes from 0 to −0.8 mA and saturates at −0.78 mA. Consequently, for subsequent experiments, we limit the current sweep to below 0.8 mA. The comparison of switching behaviors under positive and negative fields at 10 K is displayed in Fig. 2c and d. Apart from the opposite polarities, the normalized *R_A_* resistances and critical currents decrease with increasing fields because the magnetization gradually becomes fixed in the plane. In Fig. 2e, the normalized *R_A_* resistances and switching currents decrease with increasing temperature, attributable to the simultaneous decrease in magnetization. Given the semiconducting properties of MST, the resistivity of MST increases as the temperature decreases, leading to a more pronounced Joule heating effect at lower temperatures. This explains the distortion observed in the switching loop at 4 K.

To quantify the efficiency, we utilize harmonic Hall effect measurements to characterize the effective field of SOT, providing valuable insights into each SOT [38–41]. The effective SOT field **B_eff_** can be decomposed into two components: damping-like field **B_DL_** and field-like field **B_FL_**, corresponding to damping-like torque **τ_DL_** and field-like torque **τ_FL_**. An ac current, *I* = *I_0_*sin(ωt), can generate an alternating effective field **B_eff_** = **B_0_**sin(ωt), driving the magnetization **M** to oscillate around its equilibrium position, and then give rise to a second-harmonic resistance $R_{xy}^{2\omega } = - \frac{1}{2}{I_0}d{R_{xy}}/dI$. It is established both theoretically and experimentally that the anomalous Hall resistance, the planar Hall resistance (*R*_PHE_) and thermoelectric signals (*R*_T_) must be considered together to analyze the second-harmonic signals. The expressions for the first and second harmonic Hall resistances can be written as


(3) \begin{eqnarray*}
R_{xy}^\omega = {R_{AHE}}{\mathrm{cos}}\theta + {R_{PHE}}{\sin ^2}\left( \theta \right)\sin \left( {2\varphi } \right),
\end{eqnarray*}

(4) \begin{eqnarray*}
R_{xy}^{2\omega } &=& \left[ {{R_{AHE}} - 2{R_{PHE}}{\mathrm{cos}}\theta \sin \left( {2\varphi } \right)} \right]\frac{{d{\mathrm{cos}}\theta }}{{d{{\bf{B}}_{\bf{I}}}}} \cdot {{\bf{B}}_{\bf{I}}} \\
&&+ {R_{PHE}}{\mathrm{si}}{{\mathrm{n}}^2}\theta \frac{{d\sin \left( {2\varphi } \right)}}{{d{{\bf{B}}_{\bf{I}}}}} \cdot {{\bf{B}}_{\bf{I}}}\\
&&+ {R_T}{\mathrm{sin}}\theta {\mathrm{cos}}\varphi ,
\end{eqnarray*}

where *θ* and *φ* denote the polar and azimuthal angles of the magnetization vector, respectively. ${R_T}$ is the thermoelectric resistance, and **B_I_** = **B_FL_** + **B_DL_** + **B_Oe_** represents the sum of the current-induced fields, including the Oersted term. ${R_T}$ is usually a constant for the dominant anomalous Nernst effect (ANE) observed in ferromagnetic systems. When an external field **B_ext_** is applied in-plane and *θ* is set to 90°, for the ferromagnet with a perpendicular anisotropy, Equation (4) can be simplified to


(5) \begin{eqnarray*}
R_{xy}^{2\omega } &=& \left[ {\frac{{dR_{xy}^\omega }}{{d{\theta _B}}}\frac{{{B_{DL}}}}{{{B_{ext}} - {B_K}}} + \frac{{dR_{xy}^\omega }}{{d{\varphi _B}}}\frac{{{B_{FL}} \!+\! {B_{Oe}}}}{{{B_{ext}}}} \!+\! {R_T}} \right]\\
&& \times cos\varphi ,
\end{eqnarray*}

where the three terms represent the contributions of **B_DL_, B_FL_** + **B_Oe_**, and thermal effects, respectively. The anisotropy field **B_K_** is achieved from the angle-dependent AHE measurements (see Section 3). Equation (5) can be further rewritten as:


(6) \begin{eqnarray*}
R_{xy}^{2\omega } &=& \left[ \left( {{R_{AHE}}\frac{{{B_{DL}}}}{{{B_{ext}} - {B_K}}} + {R_T}} \right){\mathrm{cos}}\varphi \right. \\
&& \left. +\, 2{R_{PHE}}\left( {2{\mathrm{co}}{{\mathrm{s}}^3}\varphi - {\mathrm{cos}}\varphi } \right)\frac{{{B_{FL}} + {B_{Oe}}}}{{{B_{ext}}}} \right] \\
&=& \left[ {{R_{c1}}{\mathrm{cos}}\varphi + {R_{c2}}\left( {2{\mathrm{co}}{{\mathrm{s}}^3}\varphi - {\mathrm{cos}}\varphi } \right)} \right],
\end{eqnarray*}

where ${R_{c1}}$ and ${R_{c2}}$ are the coefficients of ${\mathrm{cos}}\varphi $ and $2{\mathrm{co}}{{\mathrm{s}}^3}\varphi - {\mathrm{cos}}\varphi $, respectively. Since the contributions of **B_DL_** and thermal are proportional to cos*φ*, these two terms can be combined into ${R_{c1}}{\mathrm{cos}}\varphi $. Similarly, the part of **B_FL_** + **B_Oe_** can be written as ${R_{c2}}( {2{\mathrm{co}}{{\mathrm{s}}^3}\varphi - {\mathrm{cos}}\varphi } )$. It is noted that there is only the cos*φ* term when φ is 45°, 135°, 225° and 315° in Equation (6), implying *R_c1_* can be obtained from these angles. Then, the $2{\mathrm{co}}{{\mathrm{s}}^3}\varphi - {\mathrm{cos}}\varphi $ term can be fitted by $R_{xy}^{2\omega } - {R_{c1}}cos\varphi $. Hence, the effective SOT fields can be extracted through the fitting of *R_c1_* and *R_c2_*.

**Figure 3.**
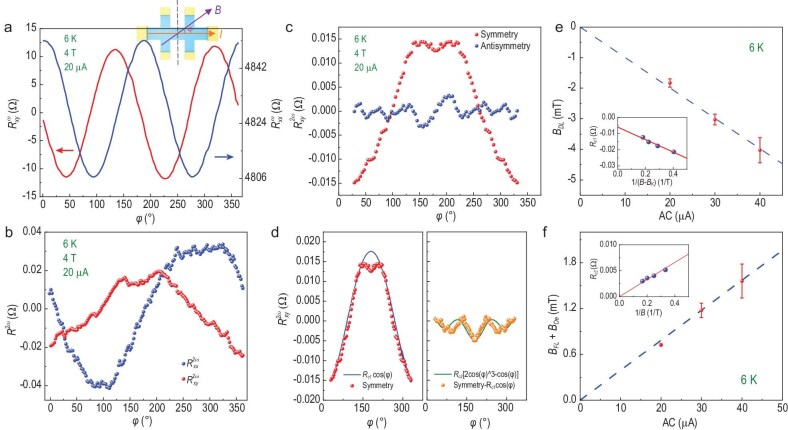
Characterization of the current-induced effective fields. (a, b) The first and second harmonic *R_xx_* and *R_xy_* as a function of the azimuthal angle φ at 6 K and 4 T. The inset is the measurement set-up. (c) The symmetric and antisymmetric components of the second-harmonic *R_xy_* at 6 K under a 4 T magnetic field, with a 20 μA ac current. (d) The fitting curves of the symmetric second-harmonic Hall resistances, showing distinct contributions of the damping-like torque (left panel) and field-like torque (right panel). (e, f) The damping-like and field-like effective fields as a function of the ac current at 6 K. The inset shows the fitting curves of *R_c1_* and *R_c2_* with a 20 μA ac current.

Here, we fix the magnitude of the magnetic field and rotate the field direction in the *x-y* plane to obtain the $R_{xy}^\omega $, $R_{xx}^\omega $, $R_{xx}^{2\omega }$ and $R_{xy}^{2\omega }$ resistance as a function of azimuthal angle φ, as shown in Fig. 3a and b. The first-harmonic and second-harmonic signals have similar phase shift behavior but with different shift angles. The raw data of $R_{xy}^{2\omega }$ at 6 K under 4 T with a 20 μA ac current have been decomposed into the symmetric and antisymmetric parts around 180° in Fig. 3c, which is crucial for excluding extrinsic signals in the antisymmetric part. Then, the *R_c1_* and *R_c2_* are separately fitted from the symmetric part, as shown in Fig. 3d. Due to the linear relationship *R_c1_* ∼ $\frac{{{B_{DL}}}}{{{B_{ext}} - {B_K}}}$ and *R_c2_* ∼ $\frac{{{B_{FL}} + {B_{Oe}}}}{{{B_{ext}}}}$, the effective SOT fields can be acquired in Fig. 3e and f. It is worth noting that the absolute value of *B_DL_* is significantly larger than *B_FL_*, which is consistent with the **τ_DL_**-dominant switching scenario depicted in Fig. 1e and f. In general, the SOT can be evaluated by the spin Hall angle formula: ${\theta _{SH}} = \frac{{2e}}{\hbar }\frac{{{M_s}t{B_{DL/FL}}}}{J},$ where *t* represents the thickness of the film, *M_S_* is the saturated moment (∼130 emu/cm^3^ at 6 K) and *J* is the ac current density. Since the Oersted field is much smaller than the two effective fields, we can calculate the damping-like torque ratio $| {\frac{{{B_{DL}}}}{J}} |$ of 1.0 × 10^−4^ mT/(A·cm^−2^) and field-like torque ratio $| {\frac{{{B_{FL}}}}{J}} |$ of 3.9 × 10^−5^ mT/(A·cm^−2^). Finally, |*θ_SH_*| can be estimated to be ∼41 for damping-like torque and ∼16 for field-like torque at 6 K, which closely resemble those reported for TI/FM systems [16,34].

## SOT SWITCHING ORIGINATING FROM THE TOPOLOGICAL BAND STRUCTURE

We further investigate the origin of SOT in MST. A pertinent question arises: why do spin accumulations not cancel in the MST system? The zero AHC and non-zero constant SHC in the band gap indicate non-trivial topological properties of the band structure, while the zero AHC illustrates that the system is not a Chern-number insulator. To identify the topological properties, we analyzed the topological surface states on different surfaces based on the surface Green's function method, as depicted in Fig. S2. No surface state is found on the two surfaces along the $[ {0001} ]$ directions, whereas the surface states connected to the valence and conduction bands are identified on the other four surfaces. The top and bottom surface states along both $[ {11\bar 20} ]$ and $[ {1\bar 100} ]$ directions exhibit slight asymmetry at $- k$ and $+ k$. This is caused by the time-reversal symmetry breaking. It usually kills one branch of the surface state in a Chern-number insulator so that quantized AHC would be identified. However, such an asymmetric effect is weak enough that two branches of the surface state at $- k$ and $+ k$ coexist on both surfaces. The spin z-component of the $+ k$ branch of the top surface is opposite to that of the $- k$ branch of the same surface and also to the $+ k$ branch of the bottom surface state, while being identical to the $- k$ branch of the bottom surface. Such topological surface states can lead to vanishing AHC and non-zero SHC within the band gap. The non-trivial topological properties are also observed in other magnetic topological systems [42,43]. Considering the low longitudinal conductivity and the high spin Hall angle when the Fermi level is inside the band gap, the large pure spin current is generated by SHC with a small electric current applied, leading to the large SOT.

**Figure 4.**
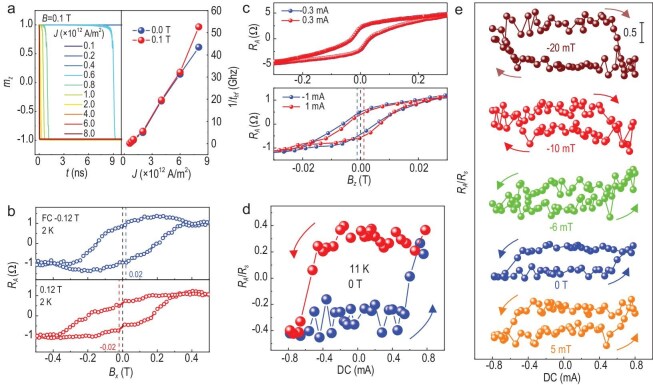
Field-free switching in MnSb_2_Te_4_/FeTe_0.9_. (a) Critical spin currents from micromagnetic simulations. The left panel shows the time-dependence of the magnetic moment by applying a magnetic field of 0.1 T. The right panel is the reciprocal of the half spin-flip time as a function of the spin current density. (b) Exchange-bias measurements after cooling the sample with ±0.12 T in-plane field. (c) AHE measurements when the dc biases are set to be ±0.3 mA and ±1 mA. (d) Field-free magnetization switching at 11 K. (e) Current-driven switching at 11 K under 5 mT, 0 mT, −6 mT, −10 mT and −20 mT. For the in-plane field −6 mT, the switching polarity remains anticlockwise, confirming the occurrence of field-free switching.

Given the large SOT effective fields observed from the experiments, we further validated the spin dynamic properties of the system through micromagnetic simulations. The polarization of the spin current aligns with the SHC results which is along the $yz$-plane. The applied magnetic field is set to 0.1T along the $y$ direction, coinciding with the direction of the applied electric current. The *z* component of the magnetization as a function of time is illustrated in the left panel of Fig. 4a. When the spin current exceeds $0.8 \times {10^{12}}( {\frac{\hbar }{e}} ){\mathrm{A}}/{{\mathrm{m}}^2},$ the initial $+ z$ magnetization can be flipped to the $- z$ direction. The right panel also displays the inverse of the half of the spin-flip time, or the spin-flip frequency, as a function of spin current under both zero and non-zero applied magnetic fields. The application of a magnetic field enhances the spin-flip frequency and reduces the critical spin current required to reverse the magnetization.

## FIELD-FREE SWITCHING IN MnSb_2_Te_4_/FeTe_0.9_

Theoretical calculations indicate the possible field-free switching in the MST system by large excitation pulses. However, due to the Joule heating effect, the amplitude of pulses has been strictly restricted. There are several reported field-free approaches for deterministic spin-orbit torque switching of the perpendicular magnet, such as lateral structural symmetry breaking [44], exchange-bias [45,46], chiral symmetry breaking [10] and so on [9,47,48]. However, many of them require complex structural designs, which might restrain the SOT effect from MST itself. Based on the theoretical analysis above, the external magnetic field can increase the spin-flip frequency and reduce the critical spin current. In FM/AFM systems, the effective field induced by the exchange bias is similar to the external magnetic field, indicating the possibility of field-free switching. This approach, utilizing exchange bias, can be easily accomplished to achieve field-free switching with a simple structure, aligning with the characteristics of the MST system.

To realize a field-free switching system, we grew a heterostructure of MnSb_2_Te_4_(8 nm)/FeTe_0.9_(4 nm) (MST/FT) on sapphire. FT is an in-plane antiferromagnetic material [49] when grown along the (001) direction, allowing for the implementation of exchange bias within the MST/FT system. Unlike MnTe, FT possesses a distinct crystal structure from MST, which helps avoid phase mixing. While FeSe presents itself as a potential antiferromagnetic candidate, the diffusion of Se atoms into MST could lead to the formation of MnSb_2_(Te_1-x_Se_x_)_4_, compromising the system's purity. Therefore, the FT is a relatively suitable antiferromagnet for our heterostructure. As shown in Fig. 4b, the exchange-bias measurements were conducted after a field-cooling process. Since the Néel temperature (*T_N_*) of FT is below 80 K, the system was firstly warmed up to 100 K for demagnetization, and then cooled down to 2 K under in-plane fields of ±0.12 T. Measurements reveal that with the positive field and negative field cooling, the AHE loops display opposite shifts of −0.02 T and 0.02 T, respectively. This behavior confirms the existence of exchange bias in the MST/FT system. Notably, the two hysteresis loops exhibit different remanent resistances at zero field, implying the various domain wall motions with opposite in-plane exchange fields. In Fig. 4c, we measured the AHE resistance under different dc biases with the magnetic field applied perpendicular to the plane. When the bias currents are set to ±0.3 mA, the two AHE loops nearly overlap without discernible differences. However, when the bias currents are increased to ±1 mA, the two AHE loops exhibit opposite shifts of ±1.2 mT, signifying the presence of the z-component effective field *H_z_*. Importantly, the absence of *H_z_* does not imply that it is zero when the current is 0.3 mA. The shift can be observed only when the torque is strong enough to overcome the intrinsic damping of the MST/FT system. As shown in Fig. S12c and d, the shifts can also be observed at bias currents of ±0.8 mA and ±0.9 mA. Another unavoidable feature present in these measurements is the shrinking of the AHE loop due to the Joule heating effect. As the current increases, the elevated temperature causes a decrease in magnetization.

The presence of *H_z_* in the MST/FT heterostructure indicates the realization of deterministic field-free magnetization switching, as illustrated in Fig. 4d. The anticlockwise switching loop, measured at 11 K, reveals two distinct states sustained at the zero current. The critical current density required for switching is determined to be 5.8 × 10^5^ A/cm^2^. Figure 4e displays the switching loop under various in-plane fields. Due to the existence of exchange bias, the switching behavior is not symmetrical around the zero field. The magnetization switches with anticlockwise polarity at 5 mT, 0 T and −6 mT, indicating a similar domain motion. The polarity flips at fields of −10 mT and −20 mT. The saturated AHE resistance at −6 mT is relatively small because of the cluttered domain orientations. Similar behaviors can also be observed at 7 K in Fig. S12e. All these results coincide with the fundamental scenario of field-free switching induced by exchange bias.

Given the similar crystal structure, MBT has attracted significant attention for its quantum properties. Exotic layer-dependent quantum phenomena, such as the quantum AHE and axion insulator state [22,23], were observed in few-layer MBT. However, the magnetic state of MBT is antiferromagnetic, which is hard to manipulate. The reported MBT/Pt system [50] has achieved SOT switching with a critical switching current of 2.6 × 10^6^ A/cm², larger than that in MST. MST with tunable magnetic states [25–29] exhibits greater potential for achieving low-power SOT switching at elevated temperatures, while MBT serves as a platform for integrating quantum state manipulation with current control.

## CONCLUSIONS

Through DFT calculations, we have successfully predicted feasible magnetization switching in MST utilizing spin-orbit torque without the necessity for external spin generator layers. Experimentally, the critical current density required for this switching is found to be 7.3 × 10^5^ A/cm^2^ at 10 K, with the SOT efficiency reaching ∼41 at 6 K, comparable to that observed in TI/FM systems. The weak asymmetry between top and bottom surface states does not lead to the disappearance of either branch, ensuring persistent non-zero spin currents. Furthermore, the construction of the MST/FT heterostructure facilitates field-free magnetization switching via the effective field induced by exchange bias. These results demonstrate a novel mechanism for effective magnetization modulation within a single MST layer and highlight the potential of advanced structures for field-free switching in spintronic devices.

## METHODS

### MBE growth

MST thin films were grown on (0001) sapphire in an ultrahigh vacuum Perkin Elmer MBE system. Prior to growth, the substrates were pre-annealed in the growth chamber at temperatures up to 600°C for 30 minutes. During the growth of MST, sapphire substrates were maintained at 350°C. For the growth of MST/FT heterostructure, the MST was first grown on the substrate, and the FT was deposited on the top of MST at 280°C. High-purity Mn (99.99%), Sb (99.999%), Te (99.9999%) and Fe (99.99%) were evaporated respectively at 686°C, 420°C, 287°C and 1070°C using conventional effusion cells. Following the growth process, the substrates were cooled down to room temperature, and a 2-nm thick Al layer was deposited as a protective cap. The Al source, with a purity of 99.999%, was evaporated at 910°C.

### Structure characterizations

Cross-section TEM samples were prepared using a focused ion beam (FEI Scios DualBeam) system. HRTEM experiments were performed using FEI Titan G2 systems to characterize the structure and chemical composition. Crystal structures were further determined by XRD (Bruker D8 Discover, Bruker Inc., Billerica, MA, USA).

### Electrical and magnetization measurements

The Hall-bar devices were fabricated through the photolithography method. Electrical measurements were conducted using the Dynacool system by Quantum Design. The dc pulse and ac currents were generated by KEITHLEY 6221. We used the ac model with dc offset to realize the reading and writing of magnetic states. Data were collected using lock-in amplifiers (Stanford Research 830 and 860, Stanford Research Systems, Sunnyvale, CA, USA) with a basic frequency of 17.171 Hz. Magnetization measurements were carried out in DC-SQUID by Quantum Design with a magnetic field up to 7 T.

### First-principles calculations of band structures of MnSb_2_Te_4_

We perform DFT-based calculations with projector-augmented wave pseudopotentials implemented in the Vienna ab initio simulation (VASP) package [51,52]. The generalized gradient approximation (GGA) in Perdew, Burke and Ernzerhof (PBE) formation is used as the exchange-correlation energy, and the Hubbard U method (U = 4.0 eV, J = 0.9 eV) is applied on Mn (3d) orbitals to include strong-correlation effects [53–55]. An energy cutoff of 520 eV is used for the plane-wave expansion throughout the calculations. The k-points are sampled on a $9 \times 9 \times 9$  ${\mathrm{\Gamma }}$-centered mesh in the Brillouin zone for self-consistent calculations. For structural relaxations, we relaxed the atoms until the Hellmann-Feynman forces were less than 1 meV/Å. The DFT-D3 method was employed to treat the van der Waals interaction [56]. After we obtained the eigenstates and eigenvalues, a unitary transformation of Bloch waves was performed to construct the tight-binding Hamiltonian in a Wannier function (WF) basis by using the maximally localized Wannier functions method implemented in the Wannier90 package [57]. The intrinsic anomalous Hall conductivity and spin Hall conductivity were calculated using the WF-based Hamiltonian based on Berry curvature with a $384 \times 384 \times 384$ k-mesh in 3D BZ. The topological edge states are calculated by the iterative surface Green's function method, as implemented in the WANNIERTOOLS package [58].

### Micromagnetic simulations

Micromagnetic simulations were performed using MuMax3 [59]. In our simulations, we take the intralayer exchange stiffness ${A_{\textit{intra}}} = 2.476 \times {10^{ - 12}}$ J/m and the interlayer exchange stiffness ${A_{\textit{inter}}} = 0.495 \times {10^{ - 12}}$ J/m, saturation magnetization ${M_s} = 1.919 \times {10^5}$ A/m, anisotropy coefficient $K = 1.481 \times {10^5}$ J/m^3^, and Gilbert damping coefficient $\alpha = 0.05$. The mesh size is $128{\mathrm{\,\,}} \times {\mathrm{\,\,}}128{\mathrm{\,\,}} \times {\mathrm{\,\,}}8$ nm^3^. All the samples are discretized into tetragonal volume elements with a size of $1{\mathrm{\,\,}} \times {\mathrm{\,\,}}1{\mathrm{\,\,}} \times {\mathrm{\,\,}}2$ nm^3^ in the simulation. The SOT driven by the intrinsic spin Hall effect is simulated based on the Slonczewski formula where the SOT is composed of the damping-like term and the field-like term, expressed as


(7) \begin{eqnarray*}
{\tau _{SOT}} &=& {\tau _{DL}} + {\tau _{FL}} = - {a_J}\left( {{\bf{m}} \times \left( {{\bf{m}} \times {{\bf{m}}_p}} \right)} \right)\\
&&- \beta {a_J}\left( {{\bf{m}} \times {{\bf{m}}_p}} \right),
\end{eqnarray*}

where ${a_J} = \frac{u}{{{M_s}d}}$ with $u = \frac{{\hbar Pj}}{{2e}}$ the magnitude of spin current caused by spin Hall effect and *d* the thickness of the magnetic film, $\beta $ is the non-adiabatic coefficient, ${\bf{m}}$ is the normalized local magnetization in the magnetic film, and ${{\bf{m}}_p}$ is the polarization vector for the spin current. We set $\beta = 0.05$. The polarization rate ($| P |$) of the spin current used in all simulations is fixed at 1.0.

## Supplementary Material

nwaf178_Supplemental_File
