# Supplementary material for: Magnetization switching by asymmetric topological surfaces
Source: Natl Sci Rev. 2025 May 9;12(7):nwaf178. doi: 10.1093/nsr/nwaf178 (PMC12218207; doi:10.1093/nsr/nwaf178)
Supplement: nwaf178_Supplemental_File [file nwaf178_supplemental_file.docx]

**Supplementary Information for**

**Magnetization Switching by Asymmetric Topological Surfaces**

Zihan Li^1#^, Sheng Pan^2#^, Shanshan Liu^1^, Yuda Zhang^1^, Ang Li^3^, Linfeng Ai^1^,

Xiaoyi Xie^1^, Xiangyu Cao^1^, Zehao Jia^1^, Xiaodong Han^3^, Jiexiang Yu^2^**^*^**, Faxian Xiu^1,4,5,6^**^*^**

^1^State Key Laboratory of Surface Physics and Department of Physics, Fudan University, Shanghai 200433, China

^2^School of Physical Science and Technology, Soochow University, Suzhou 215006, China

^3^Beijing Key Lab of Microstructure and Property of Advanced Material, Institute of Microstructure and Properties of Advanced Materials, Beijing University of Technology, Beijing 100124, China

^4^Institute for Nanoelectronic Devices and Quantum Computing, Fudan University, Shanghai 200433, China

^5^Shanghai Research Center for Quantum Sciences, Shanghai 201315, China

^6^Zhangjiang Fudan International Innovation Center, Fudan University, Shanghai 201210, China

^#^These authors contributed equally to this work

*^*^*Correspondence and requests for materials should be addressed to Faxian Xiu (E-mail: [Faxian@fudan.edu.cn](mailto:Faxian@fudan.edu.cn)) and Jiexiang Yu (E-mail: [jxyu@suda.edu.cn](mailto:jxyu@suda.edu.cn)).

**Content:**

1. **DFT calculations of MnSb_2_Te_4_**
2. **Characterizations of MnSb_2_Te_4_ films**
3. **An additional collection of magnetization switching in MnSb_2_Te_4_ thin films**
4. **Anisotropy measurements and planar Hall effect measurements**
5. **Extended discussions on SOT-induced switching behaviors**
6. **Thickness-dependent measurements**
7. **Additional insights into second-harmonic measurements and DFT calculations**
8. **Detailed information on MST/FT heterostructure**
9. **References**

**1. DFT calculations of MnSb_2_Te_4_**

In Fig. S1, σ_xy_, σ_yz_, and σ_zx_ are all zero in the gap regardless of the Mn atomic spin configurations. This can also be confirmed in the transport measurements which show the relatively small AHE signals compared to other magnetic topological insulators^1^. Similar to the constant SHC in the gap, vanishing AHC originating from the surface state is an illustration of topological properties.

There is no surface state at the bottom and top surface along $\left[ 0001 \right]$ in Figs. S2a and b. On the contrary, surface states can be found in the other four surfaces, which have also been zoomed in Figs. S2e, f, I, and j. Topological surface states exist along $\left[ 11\bar{2}0 \right]$ in Figs. S2c-f. Apparently, the surface states in different surfaces have a common feature which is the asymmetry around the Γ point in the Brillouin zone. It is caused by the time-reversal symmetry breaking, which usually kills one branch of the surface state in a Chern-number insulator so that quantized AHC can be identified. However, such an asymmetric effect is weak enough here that two branches of the surface state at $-k$ and $+k$ coexist on both surfaces. As mentioned in the main text, such topological surface states can lead to vanishing AHC and non-zero SHC within the band gap. Considering the low longitudinal conductivity and the high spin Hall angle when the Fermi level is inside the band gap, the large pure spin current is generated by SHC with a small electric current applied, leading to the large SOT.

**Figure S1.** Anomalous Hall conductivity in different configurations. (a) – (c) σ_xy_, σ_yz_, and σ_zx_ with the Mn atomic spin configurations along the $\left[ 0001 \right]$, $\left[ 10\bar{1}1 \right]$ and $\left[ 10\bar{1}0 \right]$ direction, respectively. AHC in the gap is zero.


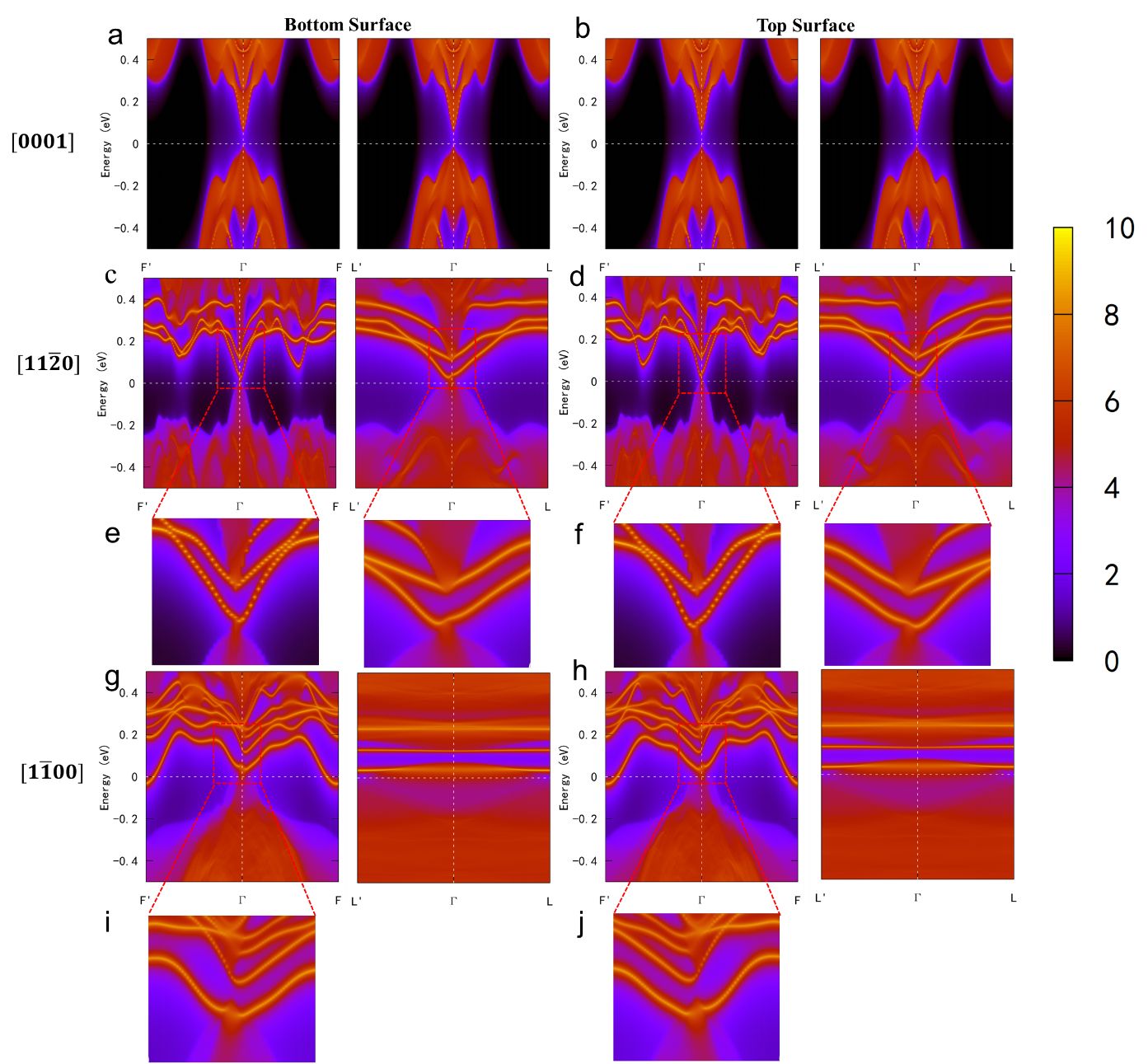


**Figure S2. Surface states for the bulk FM MST.** (a), (c), and (g) are the surface states of bottom surfaces, and each one has two calculated routes in a Brillouin zone. (b), (d), and (h) are the surface states of bottom surfaces. (e), (f), (i), and (j) are the enlarged regions near the Fermi surface. Topological surface states are displayed in (e) and (f).

**2. Characterizations of MnSb_2_Te_4_ films**

Figure S3a shows an X-ray energy dispersive spectroscopy (EDS) spectrum with element peak positions marked. The concentration ratios of Mn, Sb, and Te are 0.92:2:4, suggesting a slight deficiency in the number of magnetic Mn atoms. This deficiency leads to a lower Curie temperature of MST compared to the previous report^2^. The growth of MST is *in-situ* monitored using reflection high-energy electron diffraction (RHEED). The inset in the figure depicts sharp stripes, indicating an atomically flat surface during growth. This observation is consistent with the high crystal quality observed in TEM measurements.

The *ρ_xx_-T* curve demonstrates semiconducting behavior at low temperatures in Fig. S3b. Magnetization measurements of the ferromagnetic MST films are displayed in Fig. S3c. The inset shows a typical ferromagnetic hysteresis loop at 2 K, with a linear background attributed to the diamagnetism of the sapphire substrate. An optical micrograph of a Hall bar device is shown in Figure S3d. The device was fabricated using the photolithography process and sputtered with Au contacts. The current flows along the *x*-axis, while the channel width is 10 μm. As shown in Fig. S3e inset, *θ* is the angle between the magnetic field and the *z-*axis. Angle-dependent AHE measurements in Fig. S3e demonstrate an increase in coercive fields (*H*_c_) as *θ* varies from 0° to 90°, indicating an out-of-plane easy axis in MST. The temperature-dependent anomalous Hall effect (AHE) measurements confirm a Curie temperature of approximately 22 K, which aligns with the zero-field-cooled (ZFC) and field-cooled (FC) measurements. The DC joule heating Q=0.25 mJ can be calculated by the equation $Q=I^{2}Rt$, when the I is 0.4 mA, the R is 3165 Ω at 10 K and the t is 0.5s during one pulse.


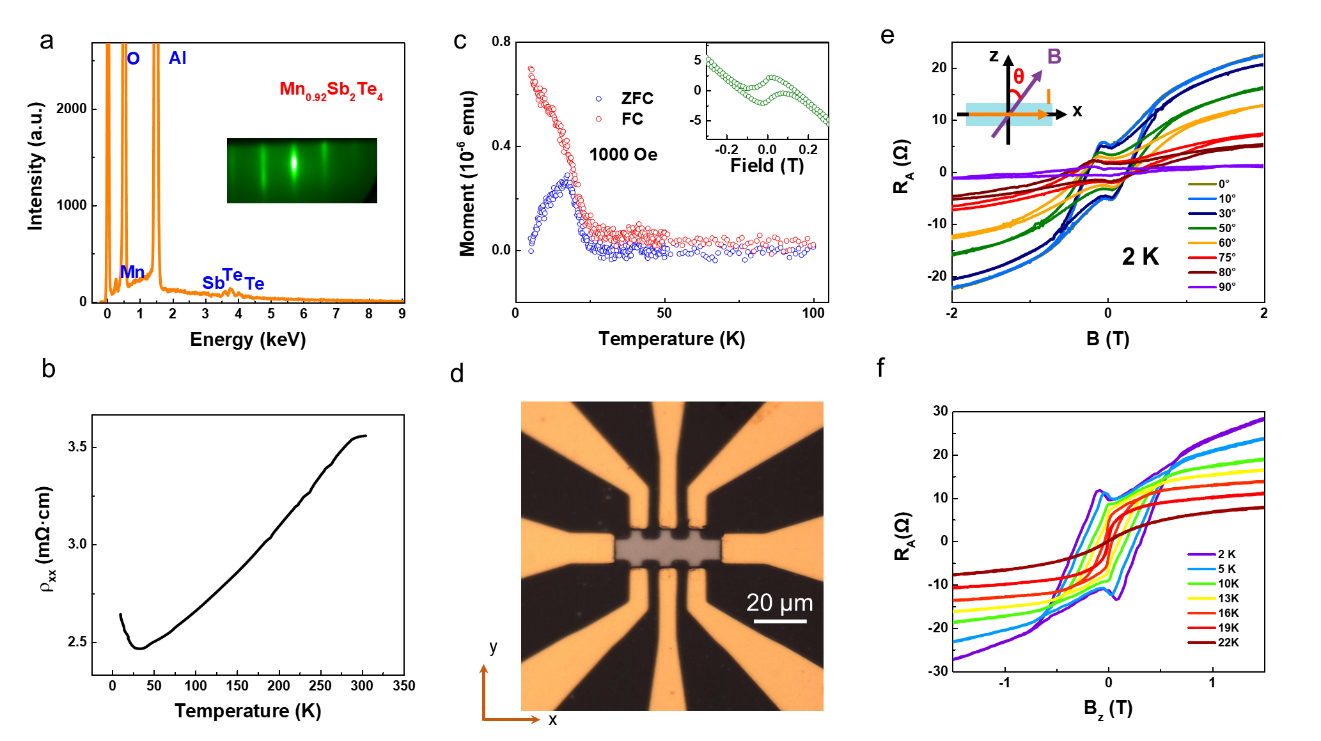


**Figure S3.** Supplementary characterizations of samples. (a) The EDS spectrum of MnSb_2_Te_4_ film with the element peak positions marked from 0 keV to 9 keV. Inset, the RHEED pattern. (b) The *ρ-T* curve of the MST film. (c) ZFC and FC magnetization curves under a magnetic field of 1000 Oe. Inset, the M-H loop at 2 K. (d) Optical micrograph of a Hall bar device used for electric measurements. The channel width of the source is 10 μm. (e) Angle-dependent AHE measurements of MST at 2 K. The inset shows the measurement geometry. The easy axis is determined to be along the out-of-plane direction. (f) Temperature-dependent AHE loops.

**3. An additional collection of magnetization switching in MnSb_2_Te_4_ thin films**

To further demonstrate various aspects of the switching behaviors, additional data of loops at temperatures ranging from 4 to 10 K are presented in Figure S4. The polarities are opposite at ±0.2 T, consistent with the loops at 14 K in the main text. As the temperature increases, the loop windows shrink due to the decrease in magnetization. As discussed in the main text, the Joule heating effect becomes more prominent as the resistance increases at lower temperatures, such as 4 K. Additionally, the resistance signals at 4 K exhibit larger noise levels. Therefore, most measurements are focused on temperatures ranging from 6 to 14 K.

To ensure repeatability and reliability, another device based on the MST film was measured. As shown in Figure S5a, two opposite AHE loops can be observed under ±0.4 mA dc bias. In comparison to the result at 10 K in Figure 1e, the *H*_c_ is much larger at 2 K despite the stronger Joule heating effect. That’s because the increasing magnetization dominates in the AHE measurements. Owing to the out-of-plane component of external fields, the saturated AHE resistances are different in the two loops. A similar dc switching loop can be seen in Fig. S5b.


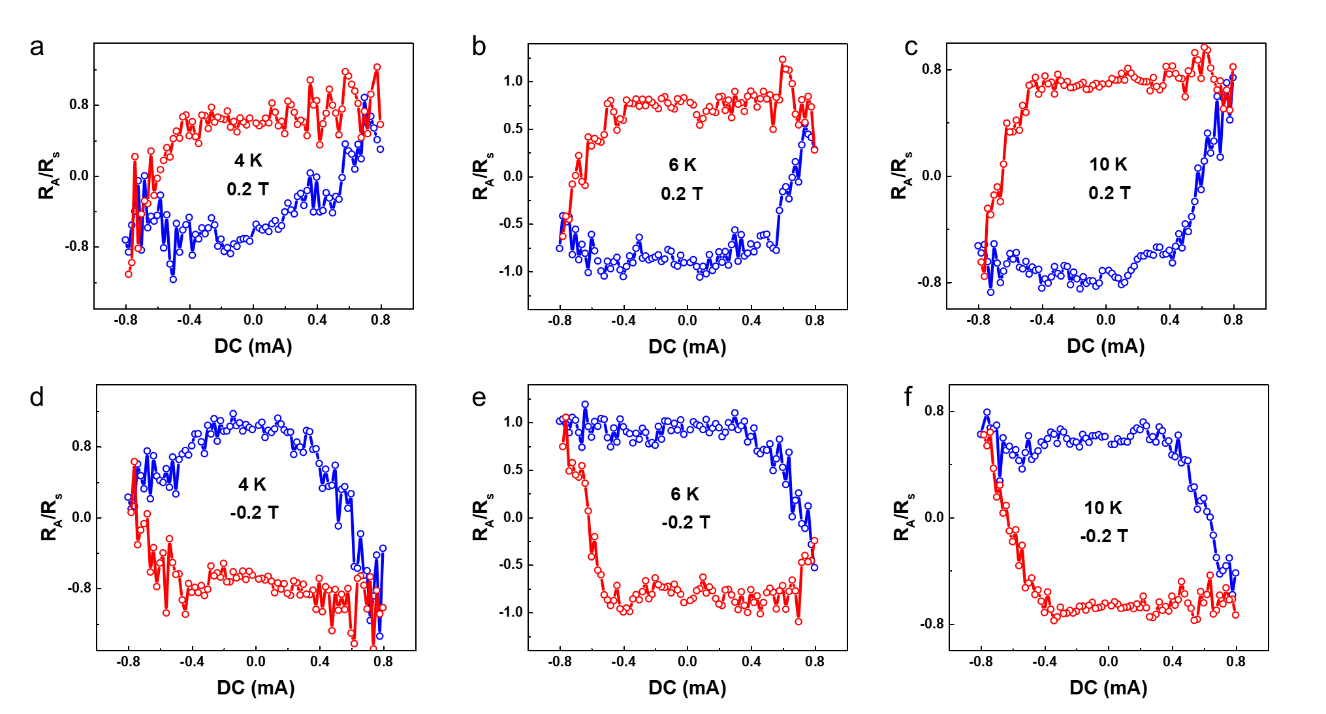


**Figure S4.** Current-driven magnetization switching under in-plane magnetic fields of 0.2 T (a)-(c) and −0.2 T (d)-(e) at 4 K, 6 K, and 10 K.


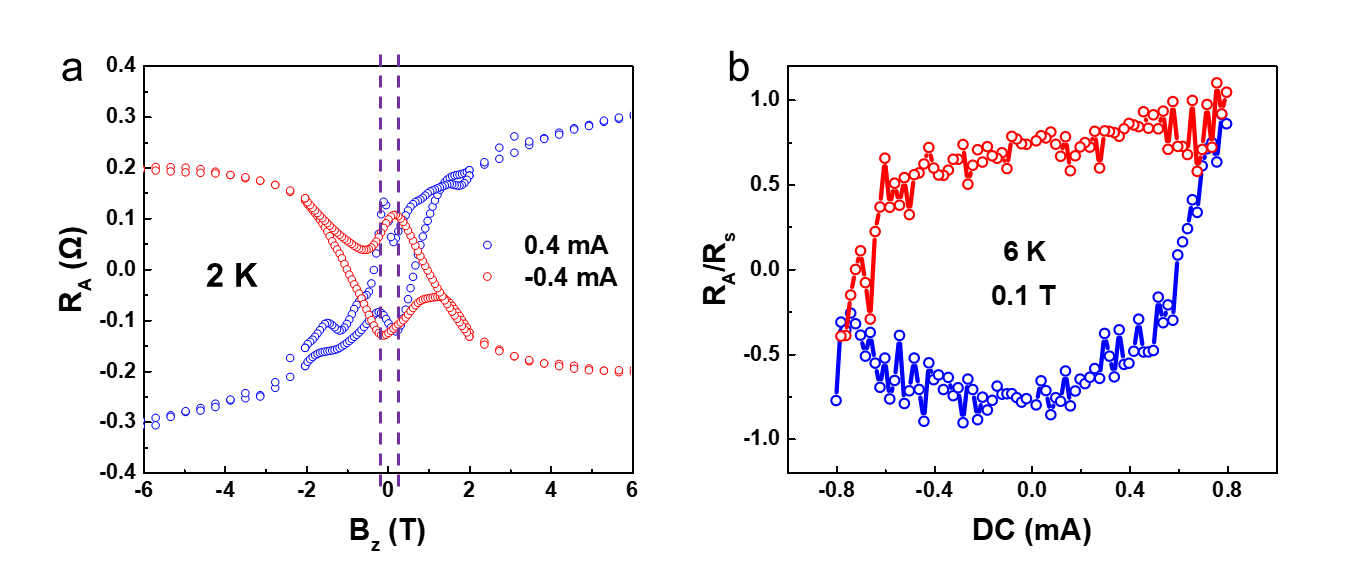


**Figure S5.** Switching data of another device based on the MST film. (a) Magnetization switching driven by a magnetic field, with ±0.4 mA dc biases at 2 K. (b) Magnetization switching induced by current at 6 K under 0.1 T.

**4. Anisotropy measurements and planar Hall effect measurements**

Angle-dependent AHE measurements were performed to calibrate the anisotropy field in MST, as shown in Fig. S6. To eliminate the domain effect, we analyzed the anomalous Hall data under a magnetic field of 6 T, where the magnetization is saturated. We start with the following function:

$$\theta_{M}\left( \theta_{B} \right)=\sin^{-1} (\frac{R_{A}\left( \theta_{B} \right)}{R_{A}\left( \theta_{B} \right)=0^{\circ}})$$

where *θ_M_* and *θ_B_* represent the angle between magnetization and the *z-*axis, and the angle between the external field and the *z-*axis. The magnetic anisotropy field is extracted by fitting *θ_M_* and*_,_ θ_H_* data with the Stoner-Wohlfarth model^3,4^. The total magnetic energy can be written as

$$E=B_{K}M_{s}\cos^{2} (\theta_{M})-BM_{s}cos(\theta_{M}-\theta_{B})$$

where *B_K_* is the anisotropy field, *M_s_* is the saturated magnetization. In the equilibrium state, $\frac{\partial E}{\partial\theta_{M}}=0$, yielding:

$$B_{K}\cos\left( \theta_{M} \right)\sin\left( \theta_{M} \right)=B\left( \sin\left( \theta_{B} \right)\cos\left( \theta_{M} \right)-\cos\left( \theta_{B} \right)\sin\left( \theta_{M} \right) \right).$$

Therefore, *B_K_* can be determined under 6 T at 6 K,10 K, and 14 K of 0.53 T, 0.60 T, and 0.47 T, respectively.

The planar Hall resistance arises from either magnetic anisotropy or the topological surface states in a TI material. This must be taken into account when analyzing second-harmonic signals. Further confirmation is observed when the planar Hall resistance is comparable to the saturated AHE resistance, as illustrated in Fig. S7.

**Figure S6.** Angle-dependent AHE at (a) 6 K, (b) 10 K, and (c) 14 K. (d) – (f) The corresponding anisotropy field analyses at 6 K, 10 K, and 14 K.

**Figure S7.** Field-dependent planar Hall effect measurements at (a) 6 K, (b) 10 K, and (c) 14 K. The applied ac current is 40 μA.

**5. Extended discussions on SOT-induced switching behaviors**

The dynamics of the magnetization subject to SOT can be described by the Landau-Lifshitz-Gilbert (LLG) equation^5^:

$$\frac{d\mathbf{m}}{dt}=-\gamma\mathbf{m}\times\mathbf{B}+\alpha\mathbf{m}\times\frac{d\mathbf{m}}{dt}+\frac{\gamma}{M_{s}}\mathbf{T}$$

where γ is the gyromagnetic ratio, **B** is the external field, α is the Gilbert damping parameter, *M_s_* is the saturation magnetization, and **m**=**M**/M_s_ is the magnetization unit vector. The SOT torque T can be written as:

$\mathbf{T}=\tau_{FL}\mathbf{m}\times\boldsymbol{\sigma}+\tau_{DL}\mathbf{m}\times\left( \mathbf{m}\times\boldsymbol{\sigma} \right)$,

where **σ** is the polarization vector along the *y*-axis. From the expression of **T**, the first term represents the part acting on the magnetization like the external field, which is called the field-like torque, while the second term acts like the damping term, which is called the damping-like torque.

Damping-like torques typically dominate in SOT devices based on TIs. The magnetization can be modulated by in-plane fields and dc currents, as illustrated in Fig. S8b. When the field and current are both along the +*x* direction, the magnetization is at the position in panel 1. When the field is along -*x* and the current is along +*x*, the magnetization shifts to panel 3. Other configurations are possible in panel 2 and panel 4. Thus, four stable magnetization states can be achieved in this scenario^6,7^.

**Figure S8.** (a) Directions of the field-like and damping-like SOT components. **σ** is along the *y*-axis. (b) Schematic of the four stable magnetization states.

**6. Thickness-dependent measurements**

Thickness-dependent measurements provide critical insights into the topological and magnetic properties of MnSb₂Te₄. We performed planar Hall effect measurements on samples with varying thicknesses, specifically at 15 nm, as illustrated in Fig. S9a. The planar Hall signal arises from both magnetic anisotropy and topological surface states, with the latter exhibiting a remarkably large amplitude^8,9^. The planar Hall effect in MST is large enough so that it cannot be ignored when it comes to the calculation of the spin Hall angle. However, the amplitude decreased with the thickness increasing. This 2D nature of the surface state confirms the topological surface states in MST. Additionally, the larger switching current density observed in the thicker sample, as shown in Fig. S9b, supports this conclusion.

We have also conducted the ZFC/FC measurements on the seven-SL sample, as shown in Fig. S9c. These measurements further confirm the ferromagnetic-paramagnetic phase transition, indicating that the interlayer coupling in our MST films is indeed ferromagnetic.


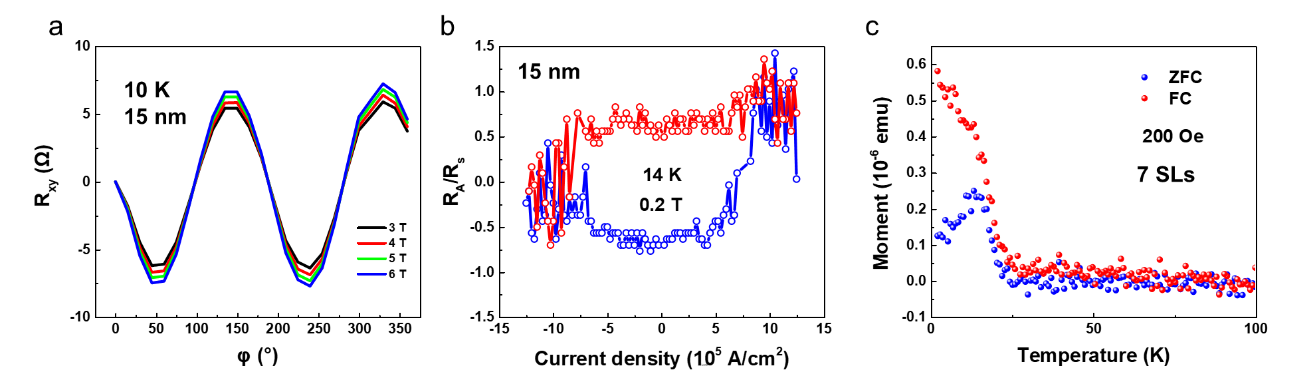


**Figure S9.** Thickness-dependent measurements. (a) Planar Hall effect measurements in 15-nm-thick MST films. (b) Current-induced switching at 14 K and 0.2 T in the 15 nm MST film. The critical current density is 8.5×10^5^ A/cm^2^. (c) ZFC/FC measurements of the MST film with 7 SLs.

**7. Additional insights into second-harmonic measurements and DFT calculations**

We use lock-in amplifiers to collect the harmonic signals generated by the constant ac current source *I*=*I_0_*sin(ωt). The Hall resistance $R_{xy}=R_{0}+\frac{IdR_{xy}}{dI}\left. \right|_{I=0}$ gives rise to the second-harmonic Hall voltage^10,11^:

$$V^{2\omega}=-\left( \frac{I_{0}^{2}}{2} \right)\left( \frac{dR_{xy}}{dI}\left. \right|_{I=0} \right).$$

When the current is sufficiently small, we obtain

$$R_{xy}^{2\omega}=V^{2\omega}/I_{0}=-\frac{1}{2}I_{0}\frac{dR_{xy}}{dI}.$$

Because **B_I_** is induced by the current *I*, we assume a linear relationship between the applied field and the current. After incorporating the thermal term, equation (2) in the main text can be expressed as:

$$R_{xy}^{2\omega}=\left[ R_{AHE}-2R_{PHE}\cos\theta\sin\left( 2\varphi\right) \right]\frac{dcos\theta}{d\mathbf{B}_{\mathbf{I}}}\cdot\mathbf{B}_{\mathbf{I}}+R_{PHE}\sin^{2}\theta\frac{d\sin\left( 2\varphi\right)}{d\mathbf{B}_{\mathbf{I}}}\cdot\mathbf{B}_{\mathbf{I}}+R_{T}\sin\theta\cos\varphi.$$

Owing to the relationship between the magnetization angles and the external field, the d**B_I_** can be divided into two orthogonal terms $dB_{I}^{\theta}=B_{ext}dsin(\theta_{B}-\theta)$ and $dB_{I}^{\varphi}=B_{ext}sin(\theta_{B})d\sin(\varphi_{B}-\varphi)$, where (*θ_B_*, *φ_B_*) is the direction of the external field. The equation can then be written as:

$$R_{xy}^{2\omega}=\left[ R_{AHE}-2R_{PHE}\cos\theta\sin\left( 2\varphi\right) \right]\frac{d\cos\theta}{d\theta_{B}}\cdot\frac{B_{I}^{\theta}}{\cos\left( \theta_{B}-\theta\right)B_{ext}}+R_{PHE}\sin^{2}\theta\frac{d\sin\left( 2\varphi\right)}{d\varphi_{B}}\cdot\frac{B_{I}^{\varphi}}{sin(\theta_{B})\cos\left( \varphi_{B}-\varphi\right)B_{ext}}+R_{T}\sin\theta\cos\varphi$$

When considering *θ_B_* = 90° and the perpendicular anisotropy, as $\frac{dR_{xy}^{\omega}}{d\theta_{B}}=R_{AHE}\frac{dcos\theta}{d\theta_{B}}$, $\frac{dR_{xy}^{\omega}}{d\varphi_{B}}=R_{PHE}\frac{d\sin\left( 2\varphi\right)}{d\varphi_{B}}$, $B_{I}^{\theta}=B_{DL}cos\varphi$, and $B_{I}^{\varphi}=B_{FL}cos\varphi$, the equation (3) in the main text can be derived:

$$R_{xy}^{2\omega}=\left[ \frac{dR_{xy}^{\omega}}{d\theta_{B}}\frac{B_{DL}}{B_{ext}-B_{K}}+\frac{dR_{xy}^{\omega}}{d\varphi_{B}}\frac{B_{FL}+B_{Oe}}{B_{ext}}+R_{T} \right]cos\varphi.$$

The anomalous Nernst effect (ANE) induced second-harmonic signal can be written as

$$R_{T}=I_{0}\beta\nabla T,$$

where $\nabla T\propto I^{2}R=\frac{1}{2}I_{0}^{2}[1-\cos\left( 2\omega t \right)]R$ is the thermal gradient, *β* is the ANE coefficient. The thermal term is a constant if the current doesn’t change in our *φ*-scan measurements.

As discussed in the main text, the raw data should be decomposed into the symmetric and antisymmetric components around 180°, as shown in Fig. 3c. Antisymmetric signals can occur due to misalignment of the sample with the external field, drift, and in-plane temperature gradients, given that the center of the Hall bar is warmer than the contact points^10^.

The fitting curve of the Oersted field and field-like effective fields are illustrated in Fig. 3f. The Oersted field is determined by Ampere's law. The *y*-oriented Oersted field is given by $B_{Oe}/J=\mu_{0}t/2$, where t = 10 nm is the thickness^12^. The Oersted field is estimated to be 6.3×10^-8^ mT/(A·cm^-2^), much smaller than effective SOT fields.

We focus on the damping-like SOT field in our MST. From the relationship $R_{c1}=R_{AHE}\frac{B_{DL}}{B_{ext}-B_{K}}+R_{T}$, we can deduce that $R_{c1}$ is linear to $\frac{1}{B-B_{K}}$. The intercepts of fitted lines represent the thermal signals, while the slopes contain the damping-like fields. As illustrated in Fig. S10, the absolute values of slopes decrease with decreasing current, indicating a reduction in effective fields. The absolute values of intercepts also decrease with the current, consistent with the origin of thermal signals. At a temperature of 10 K, the SOT efficiency declines with magnetization, and the thermal effect has a diminished influence on the second-harmonic signals due to changes in the ANE coefficient.

According to Ref.^13^, the overestimate of the SOT efficiency is from the nonlinear conduction in the 2nd-harmonic measurements, which is irrelevant to the moment oscillation induced by the SOT. From the basic schematic of the SOT torque in Fig. S8, $R_{xx}^{2\omega}$ depends on the magnetization direction and $R_{xy}^{2\omega}$ originated purely from magnetization oscillation by the damping-like torque, when we rotate the magnetic field in z-x plane. Different from the zero $R_{xx}^{2\omega}$ in Ref.^13^, $R_{xx}^{2\omega}$ and $R_{xy}^{2\omega}$ in Fig. S11 have the same order of magnitude, which is also consistent with the ideal model of the 2nd-harmonic signal induced by the damping-like torque^13^. This behavior confirms that the 2nd-harmonic *R_xx_* and *R_xy_* come from the SOT-related magnetic behavior instead of the nonlinear Hall effect induced by the magnon scattering. Therefore, the SOT-dominated harmonic transport measurements won’t be influenced by the DC or AC during the switching measurements. We have used the pulse current to conduct the switching experiments to avoid any misunderstanding.

**Figure S10.** Second-harmonic analyses of damping-like torque efficiency. The ac currents change from 40 uA to 20 uA at 6 K and 10 K.


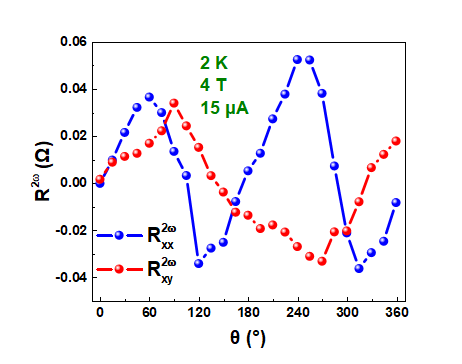


**Figure S11.** The 2nd-harmonic *R_xx_* and *R_xy_* in the z-x plane at 2 K and 4 T. They have the same order of magnitude, indicating that they come from magnetization motions induced by the damping-like torque.

**8. Detailed information on MST/FT heterostructure**

The schematic of the Hall-bar device based on the heterostructure is displayed in Fig. S12a. The channel width is 10 μm. The crystal structure has been analyzed using XRD measurements, as depicted in Fig. S12b. The characteristic peaks of MST and FT are marked with different colors. Because the MST layer is much thicker than the FT layer, the intensity of MST peaks is stronger than that of the FT peak. Moreover, the lattice mismatch can lead to an increase in the full width at half maximum of the peaks.

Additional measurements of the z-component effective field at 11 K are illustrated in Figs. S12c-d. The shifts of ±0.8 mA and ±0.9 mA dc biases correspond to ±0.6 mT and ±1.0 mT, respectively. Accompanying the decreased *H_c_* induced by the Joule heating effect, increasing the dc biases from 0.8 mA to 1.0 mA enlarges the z-component effective field from 0.6 mT to 1.2 mT. However, temperature-dependent measurements are not provided here due to the reduced diversity in the data caused by the Joule heating effect, which reduces the differences between loops at different temperatures.


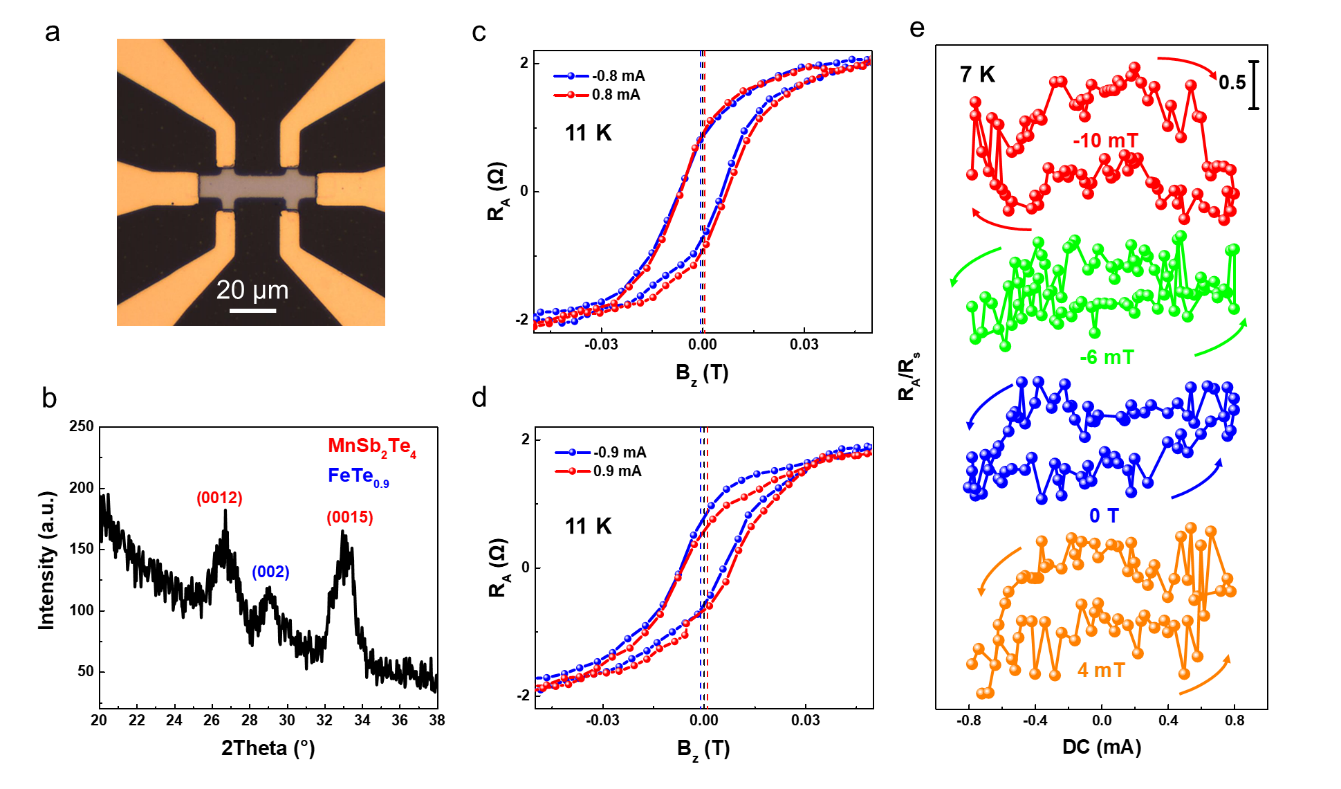
The field-free switching can also be realized at 7 K in Fig. S12e. As the temperature decreases, the noise in the data also increases. Nevertheless, the switching behavior exhibits the same characteristics as measured at 11 K. The magnetization is switched with the anticlockwise polarity at 4 mT, 0 T, and -6 mT, indicating a similar domain motion. The polarity is flipped under -10 mT. The saturated AHE resistance at -6 mT is relatively small because of the cluttered domain orientations. It should be noted that the scale bars in Figure 4e and Figure S12e are the same. It can be observed that the saturated AHE resistances at 7 K are larger than those at 11 K, which is consistent with the larger magnetization at 7 K.

**Figure S12.** Detailed information on MST/FT heterostructure. (a) An optical micrograph of a Hall bar device based on the MST/FT heterostructure. The channel width of the source is 10 μm. (b) The XRD spectrum of the heterostructure. (c) - (d) The *H_z_*-induced shifts in hysteresis loop with ±0.8 mA and ±0.9 mA. (e) Current-induced magnetization switching of MST/FT heterostructure at 7 K. The field-free switching can also be achieved.

**9. References**

1. Deng, Y. *et al.* Quantum anomalous Hall effect in intrinsic magnetic topological insulator MnBi _2_ Te _4_. *Science* **367**, 895–900 (2020).

2. Wimmer, S. *et al.* Mn-Rich MnSb2Te4: A Topological Insulator with Magnetic Gap Closing at High Curie Temperatures of 45–50 K. *Adv. Mater.* **33**, 2102935 (2021).

3. Stoner, E. C. & Wohlfarth, E. P. A mechanism of magnetic hysteresis in heterogeneous alloys. *Phil. Trans. R. Soc. Lond. A* **240**, 599–642 (1948).

4. Deng, Y. *et al.* Gate-tunable room-temperature ferromagnetism in two-dimensional Fe3GeTe2. *Nature* **563**, 94 (2018).

5. Manchon, A. *et al.* Current-induced spin-orbit torques in ferromagnetic and antiferromagnetic systems. *Rev. Mod. Phys.* **91**, 035004 (2019).

6. Fan, Y. *et al.* Magnetization switching through giant spin–orbit torque in a magnetically doped topological insulator heterostructure. *Nat. Mater.* **13**, 699–704 (2014).

7. Fan, Y. *et al.* Electric-field control of spin–orbit torque in a magnetically doped topological insulator. *Nat. Nanotechnol.* **11**, 352–359 (2016).

8. Taskin, A. A. *et al.* Planar Hall effect from the surface of topological insulators. *Nat Commun* **8**, 1340 (2017).

9. He, P. *et al.* Nonlinear Planar Hall Effect. *Phys. Rev. Lett.* **123**, 016801 (2019).

10. Avci, C. O. *et al.* Interplay of spin-orbit torque and thermoelectric effects in ferromagnet/normal-metal bilayers. *Phys. Rev. B* 11 (2014).

11. Lau, Y.-C. & Hayashi, M. Spin torque efficiency of Ta, W, and Pt in metallic bilayers evaluated by harmonic Hall and spin Hall magnetoresistance measurements. *Jpn. J. Appl. Phys.* **56**, 0802B5 (2017).

12. Wang, X. *et al.* Current-driven magnetization switching in a van der Waals ferromagnet Fe _3_ GeTe _2_. *Sci. Adv.* **5**, eaaw8904 (2019).

13. Yasuda, K. *et al.* Current-Nonlinear Hall Effect and Spin-Orbit Torque Magnetization Switching in a Magnetic Topological Insulator. *Phys. Rev. Lett.* **119**, 137204 (2017).
